# Immunomodulation by memantine in therapy of Alzheimer's disease is mediated through inhibition of K_v_1.3 channels and T cell responsiveness

**DOI:** 10.18632/oncotarget.10777

**Published:** 2016-07-22

**Authors:** Theresa Lowinus, Tanima Bose, Stefan Busse, Mandy Busse, Dirk Reinhold, Burkhart Schraven, Ursula H.H. Bommhardt

**Affiliations:** ^1^ Institute of Molecular and Clinical Immunology, Otto-von-Guericke-University Magdeburg, Magdeburg, Germany; ^2^ Molecular Physiology, Leibniz Institute for Neurobiology, Magdeburg, Germany; ^3^ Department of Psychiatry, Otto-von-Guericke-University Magdeburg, Magdeburg, Germany; ^4^ Department of Pediatric Pulmonology & Allergology, Medical University of Hannover, Hannover, Germany; ^5^ Department of Immune Control, Helmholtz Centre for Infection Research, Braunschweig, Germany; ^6^ Current address: Lee Kong Chian School of Medicine, Singapore

**Keywords:** human T cells, memantine, K_v_1.3 potassium channel, NMDA receptor antagonist, Alzheimer's disease

## Abstract

Memantine is approved for the treatment of advanced Alzheimer's disease (AD) and reduces glutamate-mediated neuronal excitotoxicity by antagonism of N-methyl-D-aspartate receptors. In the pathophysiology of AD immune responses deviate and infectious side effects are observed during memantine therapy. However, the particular effects of memantine on human T lymphocytes are unresolved. Here, we provide evidence that memantine blocks K_v_1.3 potassium channels, inhibits CD3-antibody- and alloantigen-induced proliferation and suppresses chemokine-induced migration of peripheral blood T cells of healthy donors. Concurrent with the *in vitro* data, CD4^+^ T cells from AD patients receiving therapeutic doses of memantine show a transient decline of K_v_1.3 channel activity and a long-lasting reduced proliferative response to alloantigens in mixed lymphocyte reactions. Furthermore, memantine treatment provokes a profound depletion of peripheral blood memory CD45RO^+^ CD4^+^ T cells. Thus, standard doses of memantine profoundly reduce T cell responses in treated patients through blockade of K_v_1.3 channels. This may normalize deviant immunopathology in AD and contribute to the beneficial effects of memantine, but may also account for the enhanced infection rate.

## INTRODUCTION

The non-competitive N-methyl-D-aspartate (NMDA) receptor antagonist memantine (Axura^®^, Ebixa^®^, Namenda^®^) is approved for the treatment of moderate to severe Alzheimer's disease (AD). Memantine is clinically well tolerated; it facilitates normal neuronal function due to its low affinity and fast off-rate and seems to be efficacious only under pathological conditions [1, 2]. Undesired neuronal side effects include somnolence, confusion and headache [3, 4]. Side effects on the hematopoietic and immune system have been reported with regard to infections of the respiratory and urinary tract and pancytopenia, and fungi infections were observed in 1-10 out of 1000 treated patients during post-marketing experience [5, 6].

Emerging evidence suggests that altered immune responses contribute to the pathophysiology of AD. For instance, T cell proliferation induced by amyloid beta (A-beta) or alloantigens in mixed lymphocyte reactions (MLRs) and transendothelial migration of T cells via the blood brain barrier are enhanced in AD patients compared to age-matched controls [7–13]. It was also demonstrated that an aberrant, glutamate-dependent modulation increases the activity of K_v_1.3 potassium channels on T lymphocytes of AD patients [14]. The key targets of memantine are NMDA receptors and the reduction of glutamate-mediated NMDA receptor excitotoxicity is the major mechanism whereby memantine confers neuroprotection in the treatment of advanced AD. However, NMDA receptors seem also to be expressed on non-neuronal cells, including human peripheral blood lymphocytes (PBLs) and leukemic Jurkat T cells [15–20]. In addition, memantine cross-targets other ligand-gated ion channels like K_ir_2.1 channels expressed on macrophages and microglia [21] and suppresses murine lymphocyte function through cross-inhibition of K_v_1.3 and K_Ca_3.1 potassium channels [22, 23]. The particular effects of memantine on human T cells, however, are unresolved.

Here, we provide first evidence that application of memantine blocks K_v_1.3 channel conductivity, inhibits proliferation and migration of human peripheral blood T cells and induces a strong depletion of the memory CD45RO^+^ CD4^+^ T cell pool. The data suggest that memantine's effect on human T cells suppresses adaptive immune responses and, thereby, contributes to the drug's beneficial and unwanted side effects in AD therapy.

## RESULTS

### Memantine blocks K_v_1.3 channels and suppresses the proliferation and migration of primary human T cells

**Figure 1:**
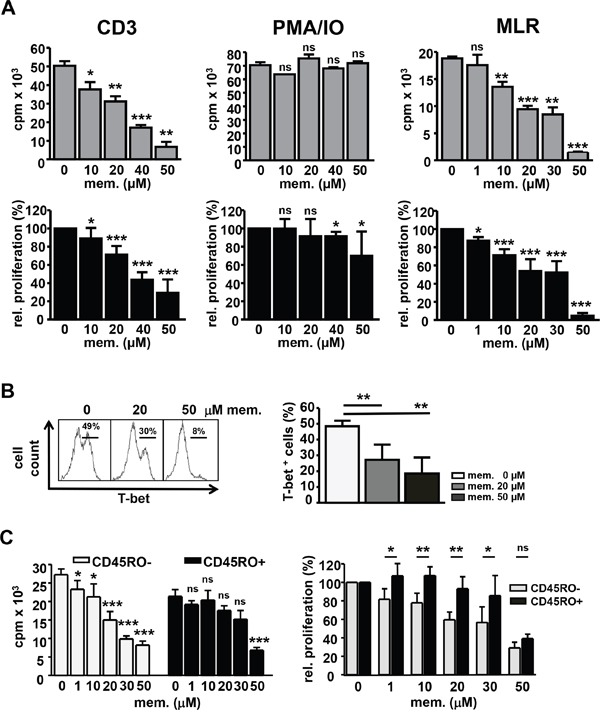
Memantine abrogates T-bet expression and proliferation of human T cells **A.** Human peripheral blood CD3^+^ T cells were stimulated with CD3 Abs (left), PMA/IO (middle) or irradiated, CD3-depleted PBMCs from another healthy donor in MLRs (right) +/- memantine. DNA synthesis was determined by ^3^[H]-Thymidine incorporation (cpm). Upper graphs represent a single experiment and lower graphs the mean relative proliferation + SD; n=4-6 and MLR n=3. **B.** T-bet expression in T cells reacting in MLRs +/- memantine was determined by flow cytometry. The histogram displays a representative experiment and data in the graph the mean + SD percentage of T-bet^+^ cells; n=5. **C.** The proliferation of naïve CD45RO^-^ and memory CD45RO^+^ CD4^+^ human T cells was analyzed in MLRs +/- memantine. Graphs show the data of a representative experiment (left) and the mean relative proliferation + SD of 5 experiments (right). The significance of data was determined with Student's *t* test; p*<0.05, p**<0.01, and p***<0.001.

To assess the impact of memantine on the proliferation of human T cells, DNA synthesis of CD3^+^ cells isolated from peripheral blood mononuclear cells (PBMCs) of healthy donors was evaluated by ^3^[H]-Thymidine incorporation in the presence or absence of memantine. Memantine inhibited CD3 Ab-induced T cell proliferation in a concentration dependent manner with an IC_50_ of ~40 μM (Figure 1A, left panel), whereas PMA/IO-activated T cells were hardly affected (Figure 1A, middle panel). Memantine doses up to 100 μM did not significantly impair the viability of human T cells (data not shown). Furthermore, we co-cultured T cells with CD3-depleted PBMCs of HLA incompatible healthy donors in MLRs. Under these more physiological stimulatory conditions, 1-10 μM memantine reduced T cell proliferation by 10-30% with an IC_50_ of ~20 μM (Figure 1A, right panel). Above results indicate that memantine's inhibitory effect on T cell proliferation inversely correlates with the strength of T cell activation.

T-bet is the critical transcription factor induced in differentiating T_H_1 cells which are responsive in alloreactive settings [24]. Using intracellular FACS staining and flow cytometry, we found that the percentage of T-bet^+^ T cells reacting to alloantigens in MLRs was profoundly diminished upon memantine treatment (Figure 1B), which substantiates memantine's suppressive effect on human T cell activation and T_H_1 cell formation.

Since memory T cells display a lower activation threshold than naïve T cells [25], we analyzed whether memantine has differential effects on those T cell subsets. In MLRs, naïve CD45RO^-^ CD4^+^ T cells were more sensitive to inhibition at lower memantine concentrations than memory CD45RO^+^ CD4^+^ T cells, which showed a significant proliferative inhibition only at 30-50 μM memantine (Figure 1C).

**Figure 2:**
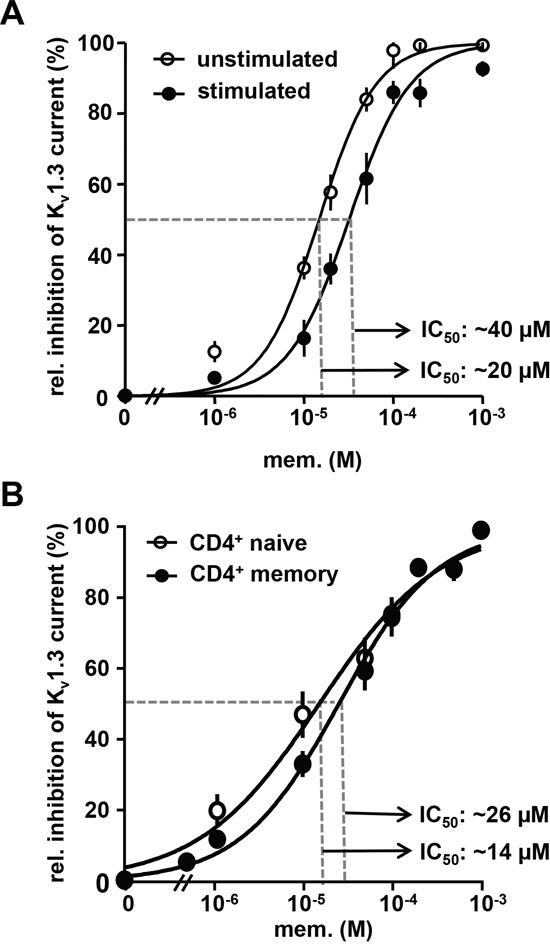
Memantine inhibits K_v_1.3 channel currents of human T cells **A.** and **B.** The dose response relationship for memantine is shown for isolated K_v_1.3 currents recorded from A. resting and CD3 Ab-activated (24 h) human CD3^+^ cells and B. naïve and memory CD4^+^ T cells isolated from peripheral blood of healthy donors. Data points represent mean values ± SEM calculated from 4-5 cells per experiment. A, n=5; B, n=4 experiments.

In murine lymphocytes memantine cross-inhibits voltage-gated K_v_1.3 potassium channels, which regulate the membrane potential and represent the driving force for Ca^2+^-influx and lymphocyte activation [22, 23, 26]. Using voltage-clamp recordings, we show here that memantine dose-dependently blocks maximal transient K_v_1.3 channel currents of primary human CD3^+^ T cells. The obtained IC_50_ values for memantine were 20 μM and 40 μM for resting and CD3 Ab-activated human T cells, and Hill slope values were 1.2 and 1.6, respectively (Figure 2A). Memory T cells express higher levels of K_v_1.3 channels than naïve T cells [27] and are less dependent on K_v_1.3 activity for IL-2 production [28]. Accordingly, memory CD45RO^+^ CD4^+^ T cells were less sensitive to inhibition of K_v_1.3 channel currents by memantine than naïve CD45RO^-^ CD4^+^ T cells, but only at lower drug concentrations (Figure 2B). Thus, memantine blocks K_v_1.3 channel currents and proliferation of both naïve and memory CD4^+^ T cells, but in line with a lower activation threshold memory CD4^+^ T cells require higher drug concentrations.

**Figure 3:**
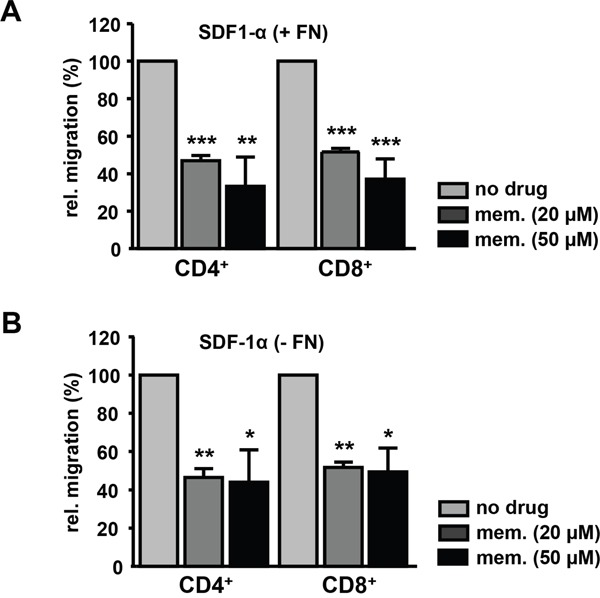
Memantine suppresses the migration of primary human T cells towards SDF-1α Isolated CD3^+^ T cells of healthy donors were left untreated or pre-incubated with memantine and their migration through **A.** fibronectin-coated (+ FN) and **B.** uncoated (- FN) transwells was induced by SDF-1α. The number of transmigrated CD4^+^ and CD8^+^ T cells was determined by flow cytometry. The graphs represent the data as mean + SD of A. 3 and B. 2 experiments. The significance of data was determined with Student's *t* test; p*<0.05, p**<0.01, and p***<0.001.

Effective immune responses depend on the migration of T cells to the sites of inflammation which is driven by chemokines like SDF-1α (CXCL12), which binds to its receptor CXCR4 expressed on T cells. Pre-treatment of human CD3^+^ cells from healthy donors with 20 μM memantine reduced SDF-1α-induced migration of CD4^+^ and CD8^+^ T cells through fibronectin-coated as well as uncoated transwells by 50% (Figure 3). The latter suggests that memantine's inhibitory effect on T cell migration is not due to a grossly altered adhesive capacity of T cells upon drug treatment. Hence, *in vitro* application of memantine inhibits K_v_1.3 channels and two important T cell responses, proliferation and migration.

### Memantine treatment of AD patients depletes memory T cells and suppresses T cell reactivity by inactivation of K_v_1.3 channels

**Figure 4:**
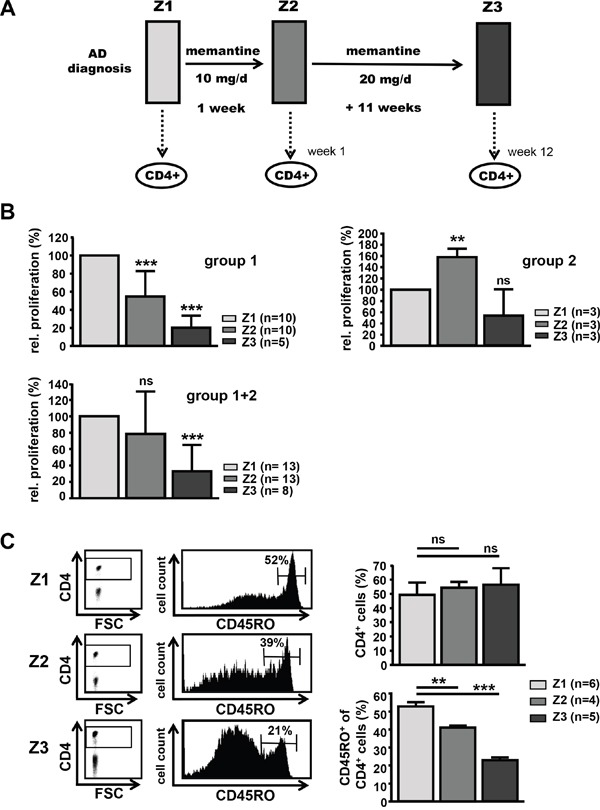
Treatment of AD patients with memantine depletes CD45RO+ CD4+ T cells and suppresses T cell responsiveness **A.** Scheme for treatment of AD patients with memantine (Axura^®^). CD4^+^ T cells of AD patients undergoing memantine therapy were analyzed at time points Z1 (after initial diagnosis and before treatment), Z2 (after one week) and Z3 (after a total of 12 weeks of daily memantine treatment). **B.** The alloresponse of CD4^+^ T cells isolated from AD patients at Z1-Z3 was determined in MLRs by ^3^[H]-Thymidine incorporation; cpm values at Z1 were set as 100%. The graphs show the mean relative proliferation + SD of T cells from two groups of patients (group 1: 5-10 patients, group 2: 3 patients) responding to memantine treatment in a different manner, and for all (8-13) patients. **C.** PBMCs of memantine-treated patients were isolated at Z1-Z3 and analyzed for the content of CD45RO^+^ CD4^+^ T cells by flow cytometry. The left panel shows representative dot plots for CD4 expression and histograms for CD45RO levels on gated CD4^+^ T cells. The right graphs display the mean percentage + SD of CD4^+^ and CD45RO^+^ cells and the number of analyzed patients. The significance of data was determined with Student's *t* test with p*<0.05, p**<0.01, and p***<0.001.

To elucidate possible side effects of memantine on adaptive immune responses during therapeutic drug administration, we studied T cell function of AD patients being treated with memantine (Axura^®^). Patients were neuro-physiologically evaluated, diagnosed, treated, and accompanied by physicians of the psychiatric department. Peripheral blood of AD patients was taken after informed consent at three time points: at Z1 before the onset of drug treatment, at Z2 after 1 week of treatment with memantine (Axura^®^, 10 mg/d) and at Z3 after additional 11 weeks of treatment with memantine (Axura^®^, 20 mg/d, i.e. in total after 12 weeks of daily drug treatment) (Figure 4A). The effect of memantine therapy on AD patients' T cell responsiveness was analyzed by alloantigen-specific T cell proliferation in MLRs. CD4^+^ T cells were isolated from the same AD individual at Z1-Z3 and co-cultured with HLA-incompatible irradiated PBMCs from the same respective healthy donor to evaluate CD4^+^ T cell reactivity of the same person to the same alloantigens before and during memantine therapy. At time point Z2, CD4^+^ T cells of most AD patients proliferated less well (10 out of 13 patients, group 1) showing a 46% reduction in DNA synthesis compared to their respective individual proliferation at Z1. For three patients CD4^+^ T cell proliferation was enhanced at Z2 (group 2). At time point Z3, alloresponses of CD4^+^ T cells from patient group 1 were further inhibited showing a 5-fold reduction of DNA synthesis compared to Z1 values. CD4^+^ T cells of patient group 2 now also showed a proliferative inhibition, but it was not significant. Considering all patients, memantine treatment led to a substantial inhibition of T cell alloreactivity with DNA synthesis being suppressed to 32% of pre-therapy values at Z3 (Figure 4B).

Since immune-pathological studies in AD patients found an increase in memory T cells compared to age-matched controls [29–31], we determined the distribution of CD45RO^+^ CD4^+^ T cells within PBMCs of AD patients at time points Z1-Z3. Whereas the percentage of total CD4^+^ T cells within PBMCs was stable, CD4^+^ T cells were significantly depleted of CD45RO^+^ cells by 22% at Z2 and by 56% at Z3 compared to Z1 pre-treatment conditions (Figure 4C). This suggests that memantine mainly affects the CD45RO^+^ T cell pool and may ‘normalize’ the pathological CD4^+^ subset composition found in AD.

**Figure 5:**
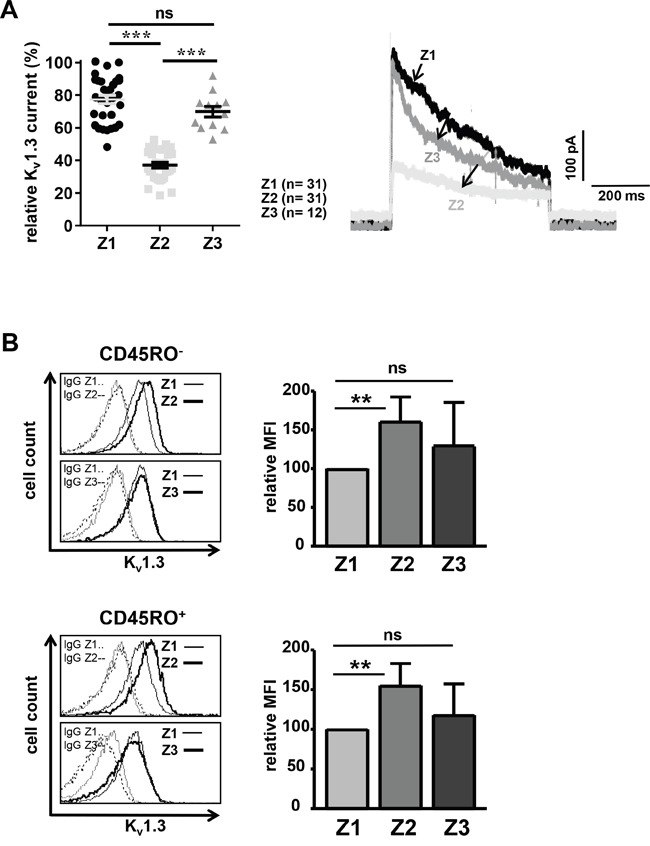
Memantine therapy transiently reduces K_v_1.3 channel conductivity of CD4+ T cells **A.** K_v_1.3 channel currents of CD4^+^ T cells from AD patients were recorded at time points Z1-Z3 by patch-clamp. Relative maximal transient currents are represented as mean ± SEM (left panel). The number of analyzed T cells is indicated. The right panel displays representative current traces at Z1-Z3. **B.** K_v_1.3 surface expression was determined on naïve (upper panel) and memory (lower panel) CD4^+^ T cells of AD patients at time points Z1-Z3 using flow cytometry; n=5 patients. Histograms show K_v_1.3 expression and IgG control staining on CD4^+^ T cells of one representative AD patient at time points Z2 and Z3 in overlay with Z1. The data in graphs give the relative mean + SD mean fluorescence intensity (MFI). MFI values at Z1 were set as 100. The significance of data was determined with Student's *t* test with p*<0.05, p**<0.01, and p***<0.001.

Given that memantine inhibits K_v_1.3 channels of human T cells of healthy donors *in vitro* (Figure 2) and a possible role of those channels in AD immune-pathogenesis [14, 21], we determined K_v_1.3 channel currents of CD4^+^ T cells from AD patients at time points Z1-Z3. Interestingly, K_v_1.3 currents at Z2 were 40% lower than those recorded at Z1, i.e. before memantine treatment, whereas at Z3 recorded K_v_1.3 currents were only reduced by 7% (Figure 5A). Thus, therapeutic application of memantine suppresses T cell function in AD patients through blockade of K_v_1.3 channel activity. Furthermore, analyzing CD4^+^ T cells of the same AD patient at Z1-Z3, we found an increased K_v_1.3 surface expression on naïve and memory CD4^+^ T cells at Z2 (60% and 55%, respectively) compared to Z1. At Z3, K_v_1.3 expression was also enhanced (by 29% and 18%), but it was not significant (Figure 5B). Thus, blockade of K_v_1.3 channel activity by memantine provokes a transient compensatory increase of K_v_1.3 channel surface expression in naïve and memory CD4^+^ T cells.

## DISCUSSION

In this report, we show that memantine suppresses the reactivity of human T lymphocytes of healthy individuals *in vitro* and of AD patients undergoing memantine (Axura^®^) therapy. The inhibitory effect of memantine on T cell proliferation directly correlated with the drug concentration and inversely with the strength of TCR stimulation, in line with the co-localization of CD3 and K_v_1.3 channels in the immunological synapse [32]. A significant inhibition of alloreactive T cell responses *in vitro* was observed with 1 μM memantine and naïve CD45RO^-^ T cells were more responsive to low memantine concentrations than memory CD45RO^+^ cells or CD3Ab-activated T cells. As memantine inhibited the induction of T-bet, which plays a pivotal role in graft rejection [24] and T_H_1 migratory programs [33], memantine may also exert potent immunosuppressive function in the prevention of transplant rejection [34, 35]. The used standard dose of memantine in the treatment of AD (10-20 mg/d) correlates with memantine serum concentrations below 1 μM [5]. T cells required much higher memantine concentrations for inhibition *in vitro*. However, standard doses of memantine given to AD patients profoundly reduced *ex vivo* T cell proliferation to 32% of initial values after 12 weeks of treatment. Thus, even low steady-state doses of memantine have substantial inhibitory side effects on T cell reactivity *in vivo* as found for higher drug concentration *in vitro*. It has also to be taken into account that in our experiments memantine was applied only once at the beginning of culture, whereas AD patients received daily medication. For immunosuppressive therapies higher drug doses of memantine may be needed, but memantine doses up to 200 mg/d seem to be tolerated concerning severe central nervous effects [5].

In line with the reported mechanism for NMDA receptor antagonists on murine T cells [22], it is conclusive that memantine significantly reduced K_v_1.3 potassium channel currents in human T cells. Compared to resting CD3^+^ and naïve CD4^+^ T cells, blockade of K_v_1.3 channels on CD3 Ab-stimulated CD3^+^ and memory T cells required higher memantine concentrations, probably due to an up-regulation of K_v_1.3 channels upon T cell activation and on memory T cells [27, 36, 37]. Memantine also suppressed T cell migration towards the chemokine SDF1-α. In view that T cell migration across the blood brain barrier is enhanced in AD patients [8], it is tempting to speculate that memantine's beneficial effect in AD treatment involves a reduced migration of inflammatory or A-beta-reactive T cells into the brain [38]. Deregulated T cell responses in the immune-pathogenesis of AD were reported to include an increased percentage of memory versus naïve T cells [29–31]. Notably, memantine given in the standard dose to newly diagnosed AD patients, substantially reduced CD45RO^+^ CD4^+^ T cells. In agreement with reports showing that K_v_1.3 channels associate with beta1 integrins [39] and are needed for effector memory T cell migration [40], the depletion of CD45RO^+^ CD4^+^ T cells may result from a reduced migration of memory cells into the blood stream or an enhanced egression.

Consistent with the *in vitro* results, K_v_1.3 potassium channel currents were significantly lower in CD4^+^ T cells of AD patients being treated with memantine for 7 days (Z2). However, the inhibition was transient as K_v_1.3 channel activity was very similar to untreated cells at 12 weeks of medication (Z3). This correlated with an enhanced surface expression of K_v_1.3 channels at Z2, probably representing a compensatory up-regulation of channel expression as a mechanism of tolerance [41]. It is important to emphasize that despite ‘restoration’ of K_v_1.3 channel activity and surface expression of K_v_1.3 channels to pre-treatment conditions at Z3, CD4^+^ T cells of most AD patients displayed progressively attenuated alloresponses at Z2 and Z3. Therefore, a transient inactivation of K_v_1.3 channel activity can correlate with a prolonged T cell unresponsiveness.

In conclusion, our data show that memantine has profound inhibitory effects on human T cells and imply that the beneficial effects of memantine in the treatment of AD may involve immunomodulation of T cells and a normalization of deviant immune functions. The drug's inhibitory side effects on T cells may also account for the enhanced infection rate during memantine application [5]. Since K_v_1.3 channel inhibitors are still quested for clinical use [42, 43], it seems worthwhile to further evaluate memantine's potential as an immune-modulating drug in the treatment of AD, autoimmune diseases or transplant rejection [44, 45].

## MATERIALS AND METHODS

### Ethics statement

Investigation has been conducted in accordance with the ethical standards and according to the Declaration of Helsinki and German guidelines and has been approved by the authors' local institutional review board (MD133/13). Written consent was obtained from all AD patients.

### Isolation of human T cells and proliferation assay

PBMCs were isolated with Biocoll Separating Solution (Biochrom AG, Berlin, Germany) and density gradient centrifugation. Isolation of CD3^+^ cells from PBMCs was performed with the human Pan T Cell Isolation Kit 2 and AutoMACS (Miltenyi Biotec, Bergisch-Gladbach, Germany). Naïve and memory CD4^+^ T cells were isolated from PBMCs with human Naïve CD4^+^ T Cell Isolation Kit 2 and human Memory CD4^+^ T Cell Isolation Kit (both Miltenyi Biotec). For patch-clamp analyses, isolation of naïve and memory CD4^+^ T cells was performed with EasySep™ Human Naïve and Human Memory CD4+ T Cell Enrichment Kits (STEMCELL, Vancouver, Canada). Purity of isolated naïve or memory CD4^+^ T cells was 80-90%. For proliferation assays, isolated T cells were cultured in AIM-V medium (Gibco, Life Technologies, Darmstadt, Germany) and stimulated with MEM92 (CD3 IgM Ab supernatant) or PMA and ionomycin (IO; each 100 ng/ml, Sigma-Aldrich, Hamburg, Germany) in the presence or absence of memantine (R&D Systems, Minneapolis, USA; Tocris Bioscience, Wiesbaden-Nordenstadt, Germany) in the indicated concentrations. 96-well plates were pre-coated with AffiniPure goat anti-mouse IgG+IgM (H+L) (Jackson ImmunoResearch Europe Ltd., Suffolk, UK) over night at 4°C, washed and incubated with MEM92 supernatant. DNA synthesis was determined in triplicates at 72 h of culture by ^3^[H]-Thymidine incorporation (0.2 μCi/ well, MP Biomedicals Europe, Heidelberg, Germany) for 16 h. For MLRs, isolated CD4^+^ T cells were stimulated with T cell-depleted and irradiated (30 Gy) PBMCs from another healthy donor in 1:3 ratios. Cells were co-cultured for 120 h and DNA synthesis was determined by ^3^[H]-Thymidine incorporation for 16 h. In MLRs, the expression of T-bet in responding T cells of healthy donors was analyzed with the intracellular staining kit (Foxp3 Staining Buffer Set, eBioscience, San Diego, USA) using T-bet-PE Ab (clone 4B10, eBioscience) and flow cytometry.

### Migration assay

Isolated CD3^+^ cells (4x10^6^) remained untreated or were pre-incubated with memantine (20 and 50 μM) for 30 min in AIM-V medium (Gibco^®^, Life Technologies) supplemented with 0.1% bovine serum albumin and 10 mM HEPES (pH 7.4) and then transferred unto uncoated or fibronectin-coated (6.5 μg/ml; Roche Diagnostics, Basel, Switzerland) transwell chambers (6.5 mm diameter and 3.0 μm pore; Corning Costar, Tewksbury, MA). Cells were allowed to migrate towards human SDF-1α (100 ng/ml; PeproTech, Hamburg, Germany) for 150 min at 37°C. Migration in the absence of chemokine served as a control. Migration was stopped by the addition of 0.1 M EDTA. Migrated cells were stained with CD4-PE and CD8-FITC Abs (BD Pharmingen, Heidelberg, Germany) and acquired for 30 sec at a FACSFortessa^TM^ (BD Bioscience, Heidelberg, Germany). Relative migration was determined by defining the number of cells that migrated in the absence of memantine (mem. 0 μM) as 100%.

### Patch-clamp analysis

Whole-cell configuration patch-clamp analysis of human peripheral blood T cells was performed as described (22). In brief, K_v_1.3 currents were recorded with an external solution containing 160 mM NaCl, 4.5 mM KCl, 5 mM HEPES, 1 mM MgCl_2_, 2 mM CaCl_2_, pH 7.4. The pipette solution was 162 mM KF, 11 mM EGTA, 10 mM HEPES, 1 mM CaCl_2_, 2 mM MgCl_2_, pH 7.2. Osmolarity was set to 300-340 mOsm. K_v_1.3 currents were measured every 30 s with depolarizing voltage steps up to +60 mV from a holding potential of -80 mV. Sampling rate during the measurement of K_v_1.3 currents was 50 kHz. The analysis of transient currents was done with HEKA FitMaster v2x53 and dose-response curves and Hill slopes were determined from amplitude values in GraphPad Prism 5.0.

### Analysis of T cells from AD patients

18 ml of blood, with heparin as anti-coagulant, was collected from AD patients for T cell proliferation and FACS analyses. In addition, routine blood analyses were performed, including determination of differential blood cell counts, levels of C-reactive protein, glucose, lipids, liver enzymes, and thyroid hormones. None of the subjects was excluded due to changes in these routine blood parameters. Also, no person had a history of autoimmune disorders, immuno-modulating treatment, cancer, chronic terminal disease, severe cardiovascular disorder, substance abuse or severe trauma. Peripheral blood samples were taken at three time points: before drug treatment (Z1), after 1 week of treatment with memantine (Axura^®^, 10 mg/d, Z2) and after 12 weeks of drug treatment (Z2 plus Axura^®^, 20 mg/d for another 11 weeks, Z3). Isolation of CD4^+^ T cells from PBMCs of patients was performed with the human CD4^+^ T Cell Isolation Kit (Miltenyi Biotec) and AutoMACS. Cells were cultured in AIM-V medium. For MLR experiments at Z1-Z3, isolated CD4^+^ T cells were co-cultured with irradiated (30 Gy) total PBMCs from healthy donors for 5 days. Proliferation of CD4^+^ T cells from the same AD patient was analyzed at Z1-Z3 to compare individual proliferation values before and after drug treatment. PBMCs from the same healthy donor were used as stimulator cells at time points Z1-Z3. DNA synthesis was determined by ^3^[H]-Thymidine incorporation at 120 h. For FACS analyses, patients' PBMCs were stained with K_v_1.3 (extracellular)-FITC (polyclonal, Sigma-Aldrich), CD45RO-PE (clone UCHL1, eBioscience) and CD4-APC Abs (clone OKT4, BioLegend, San Diego, USA) and analyzed on a FACSFortessa^TM^. K_v_1.3 expression on T cells was analyzed for the same patient at Z1-Z3. The percentage of total CD4^+^ and CD45RO^+^ CD4^+^ T cells and patch-clamp analyses at Z1-Z3 were determined on blood samples taken from different patients as drug pre-treatment values at Z1 did not significantly differ between individuals.

### Statistical analysis

Flow cytometry data were analyzed with Cell Quest Pro (Becton Dickinson, San Jose, CA, USA) or Flow Jo V3.6.1 (Tree Star, Ashland, OR, USA) software. Graphs were compiled with Graph-Pad Prism 3.0 and 5.0. Significance of data was determined with Student's *t* test with p*<0.05, p**<0.01, and p***<0.001.
